# Enhanced Anti-Tumor Activity of Cetuximab-Modified Nanostructured Lipid Carriers Loaded with *Para*-Quinone Methide Derivative *p*-QM-1h

**DOI:** 10.3390/ijms27083674

**Published:** 2026-04-20

**Authors:** Xuanze Lyu, Meijia Liu, Hanqing Li, Junyi Cui, Jie Yang, Guoyun Liu

**Affiliations:** State Key Laboratory of Macromolecular Drugs and Large-Scale Preparation, Shandong Key Laboratory of Applied Technology for Protein and Peptide Drugs, School of Pharmaceutical Sciences and Food Engineering, Liaocheng University, 1 Hunan Street, Liaocheng 252059, China

**Keywords:** nanostructured lipid carriers, cetuximab, active targeting, anti-tumor, *para*-quinone methide derivative, cell apoptosis

## Abstract

Cancer poses a serious threat to human life and health, and the number of new cancer and death cases worldwide is substantial, of which breast cancer is the most common among women. *p*-QM-1h is an organic small molecule with excellent anti-cancer activity, but it has low solubility and requires a high dosage, and it is not a targeted anti-tumor drug. In this study, *p*-QM-1h was loaded into a nanostructured lipid carrier (NLC) using the thin-film dispersion method to construct *p*-QM-1h-NLC, and its surface was modified with cetuximab (CTX) to construct CTX-*p*-QM-1h-NLC, which was tested for activity in 4T1 cells and tumor-bearing mice. The construction of CTX-*p*-QM-1h-NLC used Miglyol 812N as a liquid lipid, which effectively improved the solubility and encapsulation efficiency of *p*-QM-1h. Nanoparticles were uniform, well dispersed, and had good stability, and the CTX modification of *p*-QM-1h-NLC exhibited high connection efficiency and ensured antibody integrity. CTX-*p*-QM-1h-NLC exhibited effective anti-tumor activity in both 4T1 cells and tumor-bearing mice. The construction of CTX-*p*-QM-1h-NLC effectively improved the solubility of *p*-QM-1h, enhanced its therapeutic efficacy and reduced its drug dosage. It also had a certain targeting ability, increasing drug aggregation in tumor tissues. Flow cytometry and Western blot results showed that CTX-*p*-QM-1h-NLC could effectively inhibit the expression of TrxR and increase the expression of Bax and caspase 3 in vivo, which was consistent with the increase in ROS levels and the induction of apoptosis in 4T1 cells. These results indicated that the construction of CTX-*p*-QM-1h-NLC is worthy of further investigation.

## 1. Introduction

In 2022, there were nearly 20 million new cases of cancer worldwide, and 9.7 million people died from cancer [1]. Breast cancer and lung cancer were the most common cancers among women and men in the world, respectively, regardless of the number of new diagnoses or deaths. Female breast cancer was the second most newly diagnosed cancer in the world in 2022 and the fourth leading cause of cancer death in the world in 2022. In addition, breast cancer accounted for one-fourth of newly diagnosed cancer cases and one-sixth of cancer deaths among women worldwide. Due to the increase in aging of the global population and changes in lifestyle, it is expected that the number of new cancer cases will reach 35 million by 2050 [1]. In addition, most cancer patients are already in advanced or metastatic stages at the time of diagnosis, and existing treatment methods include surgery, chemotherapy, and a few targeted and immunotherapy treatments. With the development of medical science, molecular targeted therapy has pushed cancer treatment to a new stage [2].

Active natural products or their core structures are important sources for drug development, and more than 65% of synthetic drug molecules and 75% of anticancer drugs are derived from natural active molecules or obtained through structural optimization [3,4,5,6]. *para*-quinone methide (*p*-QM) is a class of bioactive compounds with antiviral, antifungal, antibacterial, anti-inflammatory, and antioxidant effects [7]. In our previous work, we conducted a series of structural designs on *p*-QM and studied their structure–activity relationships [8,9]. It was found that *p*-QM derivatives with *meta*-substitution had good anti-inflammatory activity. Among them, the derivative (*p*-QM-1i) substituted with trifluoromethyl could exert anti-inflammatory effects in the inflammatory cell model and the ulcerative colitis mouse model through the Toll-like Receptor 4/Nuclear factor kappa-B (TLR4/NF-κB) signaling pathway. In contrast, *ortho*-substituted derivatives had good anti-cancer effects, especially the *ortho*-trifluoromethyl-substituted *p*-QM derivative (*p*-QM-1h), which exerted anti-proliferative activity in A549 cells and anti-cancer activity in A549-tumor-bearing mice by inhibiting thioredoxin reductase (TrxR), as *p*-QM-1h contains Michael receptor units, and it had a certain degree of safety in the body. However, *p*-QM-1h is an organic small molecule with low solubility in physiological environments and requires a high dosage, and it is not a targeted anti-tumor drug.

The nanomedicine delivery system loads drugs into nanomaterials and delivers them to target tissues through specific pathways, which can improve the solubility, stability, and targeting of drugs, thereby enhancing efficacy and reducing side effects. According to material properties, nanomedicine delivery systems can be divided into lipid nanocarriers, polymer nanocarriers, inorganic nanocarriers, biomimetic or bioinspired carriers, and so on [10,11]. Nanostructured lipid carriers (NLCs) are composed of solid lipids and liquid lipids. The addition of liquid lipids can enhance drug loading capacity and is suitable for the delivery of poorly soluble drugs [12,13]. Immunoliposomes, in which antibodies are conjugated to the surface of liposomes, can be used to recognize and bind specific receptors on cells, achieving targeted delivery. The epidermal growth factor receptor (EGFR) is located on the surface of the cell membrane and is highly or abnormally expressed in malignant tumors. It is associated with the inhibition of tumor cell proliferation, angiogenesis, invasion, metastasis, and apoptosis [14]. Cetuximab (CTX) is a monoclonal antibody targeting EGFR, which plays an important role in cancer treatment by binding to EGFR to inhibit the EGFR signaling pathway, thereby suppressing tumor growth and proliferation [15,16]. Immunoliposomes conjugated with CTX can target tumor cells overexpressing EGFR, achieving efficient drug delivery [17,18].

In order to increase the bioavailability and active targeting of *p*-QM-1h and reduce the dosage and toxic side effects, we designed a nanomedicine delivery system to load *p*-QM-1h with NLC (*p*-QM-1h-NLC) and further modified the surface of *p*-QM-1h-NLC with CTX (CTX-*p*-QM-1h-NLC) to target EGFR highly expressed in tumor cells. In this study, we investigated the preparation, nano features, anti-tumor cell proliferation activity, anti-tumor activity in mice, and active targeting of CTX-*p*-QM-1h-NLC.

## 2. Results and Discussion

### 2.1. Preparation and Nano Characteristics of p-QM-1h-NLC and CTX-p-QM-1h-NLC

**Figure 1:**
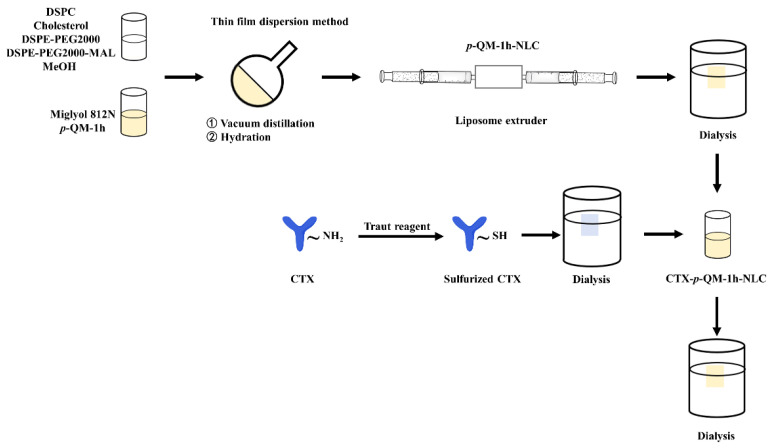
Scheme for the preparation of CTX-*p*-QM-1h-NLC.

*p*-QM-1h is an organic small molecule with low solubility. The poor water solubility and low bioavailability of drugs are the challenges facing the pharmaceutical industry [19,20]. According to the lipid ratio in Table S1, the thin-film dispersion method [21] was used to load *p*-QM-1h into NLC (Figure 1) in order to increase the solubility of *p*-QM-1h. In NLC, the regular arrangement of liquid lipids and solid lipids results in interference with each other, leading to the formation of lattice defect structures, which enables NLC to load more drugs and reduce drug leakage [22]. In the preparation of NLC, Miglyol 812N was used for liquid lipids, and DSPC, cholesterol, DSPE-PEG2000, and DSPE-PEG2000-MAL were used for solid lipids. Miglyol 812N was used as a liquid lipid for NLC to enhance drug solubility. The use of DSPE-PEG2000 can provide a hydrophilic barrier, reduce non-specific adsorption, and prolong in vivo circulation time. DSPE-PEG2000-MAL provides maleimide sites for connection with CTX. As shown in Table S2, the solubility of free *p*-QM-1h in PBS was only 14.54 ± 0.60 μg/mL. However, when Miglyol 812N was used as the liquid lipid, loading free *p*-QM-1h with NLC could greatly increase its solubility, which would be beneficial for increasing bioavailability and reducing the volume of intravenous injection in animal experiments. In addition to Miglyol 812N, oleic acid, MCT, and castor oil were also selected as liquid lipids. As shown in Tables S2 and S3, when oleic acid was selected, the solubility of *p*-QM-1h and the encapsulation efficiency (EE) of NLC were lower. When using MCT and castor oil, the solubility of *p*-QM-1h and the EE were higher; however, the purchase cost of MCT was higher, and NLC solution prepared using castor oil as liquid lipid would precipitate after storage at 4 °C for 24 h.

**Figure 2:**
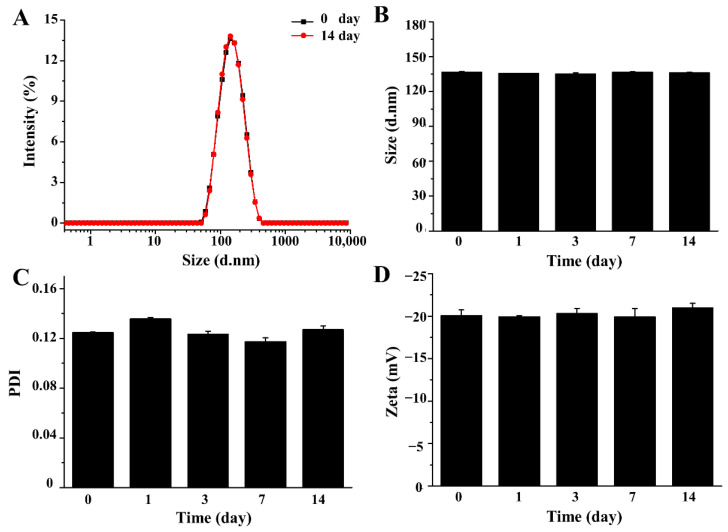
The stability testing of *p*-QM-1h-NLC. (**A**) The particle size distribution of *p*-QM-1h-NLC on day 0 and day 14. (**B**) Changes in particle size within 14 days. (**C**) Changes in PDI within 14 days. (**D**) Changes in Zeta potential within 14 days. The experiment was performed at least in triplicate. Error bars represent mean ± SD.

The nano characteristics of *p*-QM-1h-NLC were identified. As shown in Table 1, the particle size of *p*-QM-1h-NLC was 136.77 ± 0.52 nm, the PDI was 0.125 ± 0.001, and the Zeta potential was −20.07 ± 0.67 mV, which indicated that the nanoparticles were uniform, well dispersed, and had moderate stability. Furthermore, the intensity and number distributions of *p*-QM-1h-NLC were unimodal, with the peak value of number distribution slightly smaller than the peak value of intensity distribution, which also confirmed that *p*-QM-1h-NLC was uniform and without aggregates (Figure S1). The EE of *p*-QM-1h-NLC was 82.80 ± 1.48%, and the drug loading content (DLC) was 7.37 ± 0.13%, indicating that *p*-QM-1h-NLC could provide sufficient space to effectively load *p*-QM-1h. In addition, after 14 days of stability testing, the particle size, PDI, and Zeta potential of *p*-QM-1h-NLC remained basically unchanged, indicating good stability (Figure 2).

**Figure 3:**
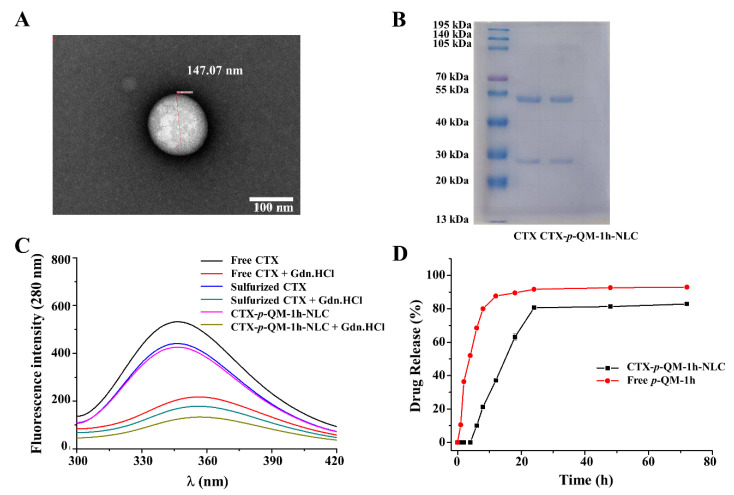
(**A**) Representative TEM image of CTX-*p*-QM-1h-NLC, (**B**,**C**) analyses of primary and tertiary protein structures of CTX, and (**D**) the drug release of CTX-*p*-QM-1h-NLC. The experiment was performed at least in triplicate. The red dashed line in subfigure A represents the diameter of the nanoparticle.

*p*-QM-1h is a non-selective anti-tumor drug, so in order to achieve active targeting of tumors, the surface of *p*-QM-1h-NLC was coupled with CTX, (Figure 1) which targeted EGFR highly expressed in tumors. CTX reacted with Traut to obtain thiolated CTX, and the SH of thiolated CTX interacted with the reserved maleimide sites on the NLC surface, originating from DSPE-PEG2000-Mal with a total lipid molar ratio of 0.1%. As shown in Table 1 and Figure S1, the particle size of CTX-*p*-QM-1h-NLC was 150.53 ± 0.68 nm, PDI was 0.123 ± 0.012, and Zeta potential was −19.07 ± 0.25 mV, which indicated that the nanoparticles of CTX-*p*-QM-1h-NLC were also uniform, well dispersed and stable. Also, the particle size of CTX-*p*-QM-1h-NLC was slightly larger than that of *p*-QM-1h-NLC or CTX-NLC. In theory, CTX connected to the surface of *p*-QM-1h-NLC acted as a shield, which could lead to a slight decrease in the absolute value of the Zeta potential of CTX-p-QM-1h-NLC. However, the amount of DSPE-PEG2000-Mal only accounted for 0.1% of the total lipids, and the density of CTX connected to the surface of CTX-p-QM-1h-NLC was relatively low; therefore, the Zeta potential change between *p*-QM-1h-NLC and CTX-p-QM-1h-NLC was not significant. Further transmission electron microscopy (TEM) analysis showed that the CTX-*p*-QM-1h-NLC nanoparticles were almost spherical in shape, with a particle size similar to that observed in the DLS results (Figure 3A and Figure S2).

Fourier transform infrared (FTIR) spectroscopy was used to investigate the interaction between *p*-QM-1h and lipids. As shown in Figure S3, the FTIR spectra of *p*-QM-1h, CTX-*p*-QM-1h-NLC, CTX-NLC and physical mixtures of *p*-QM-1h, CTX and liquids were compared. The spectrum of *p*-QM-1h had a characteristic peak at approximately 1615 cm^−1^, which also appeared in the FTIR spectra of CTX-*p*-QM-1h-NLC and the physical mixture. The spectra of CTX-NLC, CTX-*p*-QM-1h-NLC and the physical mixture had the characteristic peak at approximately 1745 cm^−1^. In these spectra, there were no new peaks or peak displacements. These data indicated that *p*-QM-1h was only physically embedded without the formation of covalent bonds, which could ensure the stability of *p*-QM-1h in NLC.

### 2.2. Immune Characteristics of CTX-p-QM-1h-NLC

In addition to performing nano characteristics detection on CTX-*p*-QM-1h-NLC, we also evaluated the connection efficiency (CE) and antibody integrity of CTX. Compared with other literature [23], the molar ratio of thiolated CTX to DSPE-PEG2000 Mal decreased to 1.5:1 [13], and under these conditions, the CE of CTX reached 87.98 ± 3.68% (Table 1).

Furthermore, in order to determine the antibody integrity of CTX attached to the surface of CTX-*p*-QM-1h-NLC, the protein primary and tertiary structures were determined. As shown in Figure 3B, both free CTX and the CTX connected on the surface of CTX-*p*-QM-1h-NLC showed two bands on the electrophoresis gel, heavy chain and light chain. This indicated that the CTX connected to the surface of CTX-*p*-QM-1h-NLC maintained the integrity of the CTX primary structure without damage. As shown in Figure 3C, under excitation at 280 nm, free CTX exhibited the maximum emission wavelength at 346.67 ± 0.12 nm due to the presence of aromatic amino acids in the protein structure [24,25]. When guanidine hydrochloride (Gdn·HCl) was added, the fluorescence spectrum of free CTX underwent a spectral shift (λ_max_ = 355.67 ± 0.31 nm) and significant fluorescence intensity suppression. The maximum emission wavelengths of thiolated CTX and the CTX coupled to the surface of CTX-*p*-QM-1h-NLC were 346.27 ± 0.23 and 346.0 ± 0.20 nm, respectively. After adding Gdn·HCl, the maximum emission wavelengths shifted to 356.2 ± 0.60 and 356.2 ± 0.42 nm, which were basically unchanged compared to the data of the free CTX sample group. This indicated that after two steps of thiolation and coupling, the tertiary structure of CTX coupled to the surface of *p*-QM-1h-NLC did not undergo significant changes and would not affect the biological function of CTX.

### 2.3. In Vitro Drug Release of CTX-p-QM-1h-NLC

The slow release characteristics of nanomedicine are one of the core advantages that distinguish nanocarriers from free drugs. The slow release of nanomedicine can prolong the duration of drug action, maintain effective drug concentration, and reduce the frequency of administration. Therefore, we measured the in vitro drug release of CTX-*p*-QM-1h-NLC in PBS containing 1% Tween-20. As shown in Figure 3D, the free *p*-QM-1h was released in the first hour and lasted for approximately 8 h; and from the eighth hour onwards, the drug release entered a plateau phase. CTX-*p*-QM-1h-NLC was released at the 6th hour and lasted for approximately 19 h. Starting from the 24th hour, drug release entered a plateau phase. The drug release time of CTX-*p*-QM-1h-NLC was 13 h longer than that of free *p*-QM-1h, indicating that CTX-*p*-QM-1h-NLC could effectively prolong the release time of *p*-QM-1h. However, it could not be ignored here that the PBS containing 1% Tween-20 was only a simple system that was homogeneous, cell-free and barrier free, and it was not a physiological environment, differing greatly from the complex microenvironment in the body.

### 2.4. Anticancer Activity of CTX-p-QM-1h-NLC in 4T1 Cells

The 4T1 cell line, a typical cell line in breast cancer research, was used to test the effects of CTX-*p*-QM-1h-NLC on cell proliferation, reactive oxygen species (ROS), mitochondrial membrane potential (MMP) and cell apoptosis of 4T1 cells.

**Figure 4:**
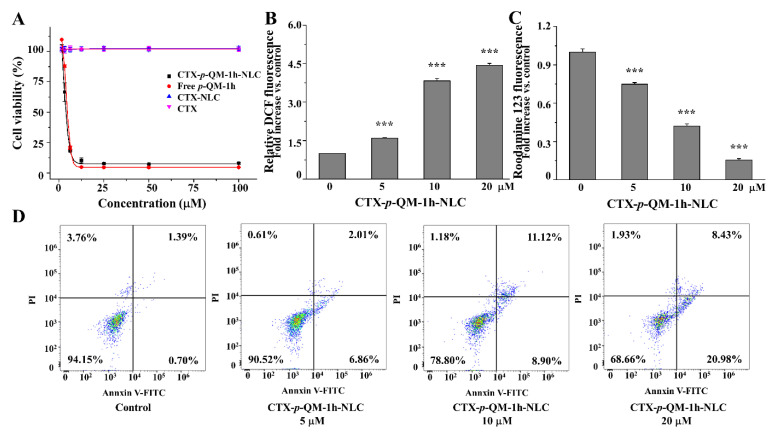
Anticancer activity of CTX-*p*-QM-1h-NLC in 4T1 cells. (**A**) Anti-proliferative activity. (**B**) ROS levels. (**C**) MMP levels. (**D**) Cell apoptosis. The experiment was performed at least in triplicate. Error bars represent mean ± SD. *** *p* < 0.001 compared with the model group.

The MTT assay was used to test the anti-proliferative activity of the drug in 4T1 cells, as shown in Table S5 and Figure 4A. The anti-proliferative activity of free *p*-QM-1h was similar to that of CTX-*p*-QM-1h-NLC; therefore, loading free *p*-QM-1h into CTX-*p*-QM-1h-NLC did not affect the anti-proliferative activity of *p*-QM-1h in 4T1 cells. The CTX-NLC and CTX did not contribute to the anti-proliferative activity of *p*-QM-1h.

Furthermore, we tested the effects of CTX-*p*-QM-1h-NLC on ROS, MMP and cell apoptosis. As shown in Figure 4B–D, CTX-*p*-QM-1h-NLC could also increase ROS levels and cause MMP breakdown in 4T1 cells, leading to cell apoptosis. This corresponded to our previous article [9], where free *p*-QM-1h with Michael receptor acted on TrxR in the cellular redox system, causing an imbalance of intracellular redox, an increase in ROS levels, and a decrease in MMP, ultimately leading to cell apoptosis.

### 2.5. In Vivo Anti-Tumor Activity

**Figure 5:**
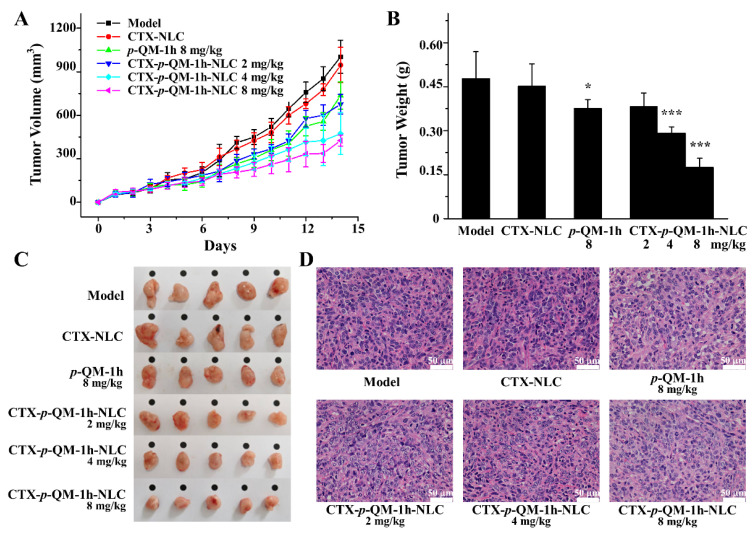
The anti-tumor activity of CTX-*p*-QM-1h-NLC in vivo (*n* = 5). (**A**) Tumor volume changes. Error bars represent mean ± SD. (**B**) Tumor weight. Error bars represent mean ± SD. * *p* < 0.05 and *** *p* < 0.001 compared with the model group. (**C**) Tumor images. (**D**) Representative images of tumor histopathology (scale bar, 50 μm).

To determine the anti-tumor activity in vivo, 4T1 tumor-bearing Balb/c mice were established as the mouse tumor model. After subcutaneous injection of 4T1 cells into mice, the tumor volume increased day by day. In Figure 5A–C, CTX-*p*-QM-1h-NLC could effectively inhibit tumor growth in mice in a dose-dependent manner. The inhibitory effect of 2 mg/kg CTX-*p*-QM-1h-NLC on tumors was similar to that of 8 mg/kg free *p*-QM-1h (Figure 5A–C), which indicated that CTX-*p*-QM-1h-NLC could effectively enhance the anti-tumor therapeutic effect; moreover, under the same therapeutic effect, the lower dosage of CTX-*p*-QM-1h-NLC was beneficial for reducing toxicity and improving safety. Non-drug-loaded CTX-NLC had no significant tumor suppressive effect, which may be due to the very low dosage of CTX (1.55 mg/m^2^), far below the recommended dosage for CTX (initial dosage 400 mg/m^2^ and maintenance dosage of 250 mg/m^2^). (Please refer to the Supporting Information for the specific calculation process.) This indicated that CTX did not contribute to the improvement of drug efficacy and was only used for targeting purposes; this also suggested that the improvement in drug efficacy was due to the increased solubility of *p*-QM-1h loaded into CTX-*p*-QM-1h-NLC or the targeting effect of CTX.

For the histopathological analysis of tumor tissue slices, as shown in Figure 5D, the tumor slices of the model group showed that the cells were arranged neatly, whereas the tumor slices of the CTX-*p*-QM-1h-NLC treatment group showed significant nuclear shrinkage. This indicated that the intervention of CTX-*p*-QM-1h-NLC caused damage to tumor tissue.

In vitro studies have shown that CTX-*p*-QM-1h-NLC could lead to an increase in intracellular ROS levels and MMP breakdown, and trigger cell apoptosis in 4T1 cells. Moreover, as reported in our previous work [9], *p*-QM-1h containing the Michael receptor unit could covalently react with the highly expressed TrxR in tumor cells to inhibit TrxR activity, thereby disrupting the redox balance in tumor cells, increasing intracellular ROS levels, and inducing tumor cell apoptosis. Therefore, we further performed Western blot analysis on TrxR and apoptosis-related proteins (Bax and Caspase-3) in the 4T1 tumor-bearing Balb/c mice in vivo.

TrxR is a differential protein between tumors and normal cells and is much higher in various human tumor tissues than in normal human tissues [26,27]. TrxR is susceptible to covalent reactions with electrophilic reagents containing Michael receptor units, inhibiting TrxR expression, disrupting the redox balance of tumor cells, increasing intracellular ROS levels, breaking the “death threshold” of ROS, and inducing cell apoptosis, thereby exerting anti-tumor effects [28].

**Figure 6:**
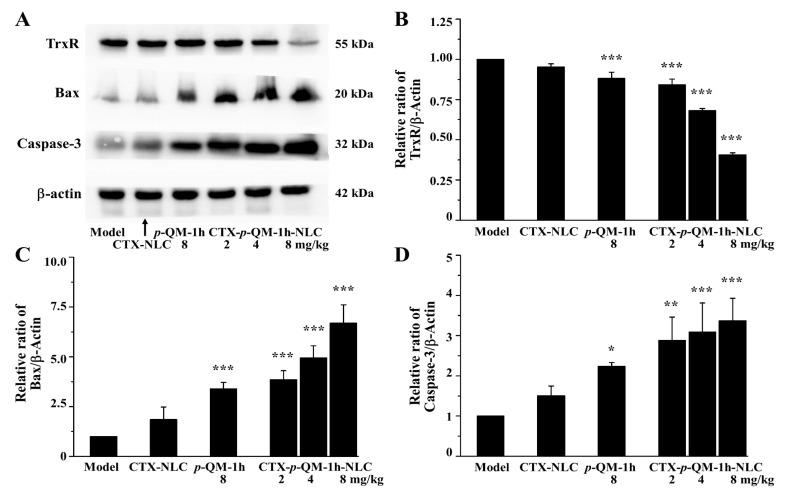
The effects of CTX-*p*-QM-1h-NLC on the expressions of TrxR, Bax and Caspase-3 in tumors of 4T1 tumor-bearing Balb/c mice. (**A**) Representative target protein bands. The experiment was performed at least in triplicate. (**B**) Relative ratio of TrxR. (**C**) Relative ratio of Bax. (**D**) Relative ratio of Caspase-3. The grayscale quantitative analyses of the expressions of TrxR, Bax and Caspase-3 were performed using the ImageJ software (1.53e). Error bars represent mean ± SD. * *p* < 0.05, ** *p* < 0.01 and *** *p* < 0.001 compared with the model group.

As shown in Figure 6A,B, compared with the expression level of TrxR in the tumor tissue of the model group, the treatment of tumor mice with 8 mg/kg *p*-QM-1h resulted in a decrease in TrxR levels; the treatment of tumor mice with CTX-*p*-QM-1h-NLC also resulted in a decrease in TrxR levels in a dose-dependent manner. Moreover, the therapeutic effect of 2 mg/kg CTX-*p*-QM-1h-NLC was similar to that of 8 mg/kg *p*-QM-1h. The TrxR level of the CTX-NLC treatment group was equivalent to that of the model group.

For proteins related to cell apoptosis [29], the activation of Bax is a key initiating event in the apoptotic cascade, and Bax has pro-apoptotic activity; Caspase-3 is the terminal effector protease of the cell apoptosis signaling pathway, and its activation is a sign of irreversible execution of apoptosis.

As shown in Figure 6A,C,D, compared with the expression levels of Bax and Caspase-3 in tumor tissues of the model group, the drug treatment groups increased these expression levels. The expression levels of Bax and Caspase-3 in the CTX-NLC treatment group were comparable to those in the model group.

This indicated that the treatment with CTX-*p*-QM-1h-NLC could effectively inhibit TrxR and increase the expressions of Bax and Caspase-3, which was consistent with the increases in ROS levels and cell apoptosis in the cell system. Moreover, loading *p*-QM-1h into CTX-*p*-QM-1h-NLC could effectively enhance the efficacy of free *p*-QM-1h.

### 2.6. In Vitro and In Vivo Targeting Study of CTX-p-QM-1h-NLC

The targeting ability of nanomedicine is one of its core advantages. Targeted nanomedicine can be specifically enriched in the lesion site, significantly increasing the concentration of the drug in the lesion tissue. Moreover, targeted nanomedicine can prevent the systemic distribution of free drugs from damaging normal tissues, thereby reducing damage to normal tissues. In addition, high targeting also means lower dosage and further reduces toxic side effects. The targeting study of a nanomedicine is a key step in evaluating its therapeutic efficacy.

EGFR is highly or abnormally expressed in many malignant tumors and is a target for tumor treatment. According to literature reports [30,31], Western blot experiments clearly detected the expression of total EGFR protein in 4T1 cells, and immunohistochemistry and immunofluorescence staining also showed positive expression of EGFR in in situ tumors and lung metastases formed by 4T1 cells. Moreover, EGFR-targeted nanomedicines have been designed and used to treat 4T1 tumor-bearing mice [32,33,34]. Therefore, we further investigated the targeting properties of CTX-*p*-QM-1h-NLC in 4T1 cells and 4T1 tumor-bearing mice.

#### 2.6.1. In Vitro Targeting Study of CTX-p-QM-1h-NLC

**Figure 7:**
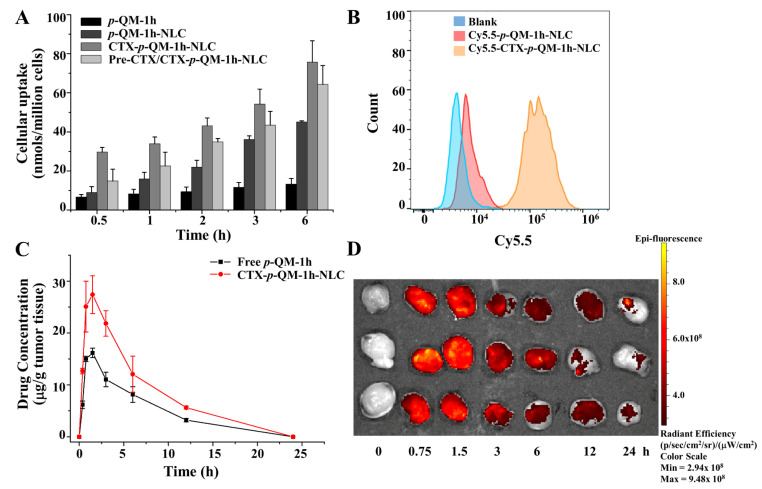
In vitro and in vivo targeted study of CTX-*p*-QM-1h-NLC. (**A**) Cellular uptake of *p*-QM-1h, *p*-QM-1h-NLC and CTX-*p*-QM-1h-NLC (pretreated with/without CTX) in 4T1 cells. The experiment was performed at least in triplicate. Error bars represent mean ± SD. (**B**) In vitro targeted study of Cy5.5-*p*-QM-1h-NLC and Cy5.5-CTX-*p*-QM-1h-NLC in 4T1 cells using the flow cytometer. (**C**) Quantitative measurements of the concentration of *p*-QM-1h and CTX-*p*-QM-1h-NLC in tumor tissues at different time points using HPLC. The experiment was performed at least in triplicate. Error bars represent mean ± SD. (**D**) Fluorescence images of Cy5.5-CTX-*p*-QM-1h-NLC in tumor tissues using the vivo imaging system.

CTX can target EGFR on the surface of cancer cells. To verify that CTX-*p*-QM-1h-NLC and CTX-NLC could target 4T1 cells, we conducted in vitro targeting studies using cellular uptake and flow cytometry. As shown in Figure 7A, the cellular uptake of *p*-QM-1h was compared among CTX-*p*-QM-1h-NLC, *p*-QM-1h-NLC or *p*-QM-1h-treated 4T1 cells. At different time points, the order of the cellular uptake of *p*-QM-1h in different drug-treated groups was as follows: CTX-*p*-QM-1h-NLC > *p*-QM-1h-NLC > *p*-QM-1h group, which indicated that nanoparticles (CTX-*p*-QM-1h-NLC and *p*-QM-1h-NLC) could significantly promote the entry of *p*-QM-1h into cells. For the CTX-pretreated group (the pre-CTX/CTX-*p*-QM-1h-NLC group), the cellular uptake level in the pre-CTX/CTX-*p*-QM-1h-NLC group was lower than that in the CTX-*p*-QM-1h-NLC group, which indicated that CTX-*p*-QM-1h-NLC increased the cellular uptake level by targeting EGFR, and also suggested that the CTX attached to the surface of *p*-QM-1h-NLC was not only structurally intact but also functionally preserved.

As shown in Figure 7B, the targeting of Cy5.5-CTX-*p*-QM-1h-NLC and Cy5.5-*p*-QM-1h-NLC on 4T1 cells was tested using flow cytometry. High fluorescence levels were detected in the 4T1 cell group treated with Cy5.5-CTX-*p*-QM-1h-NLC; however, low levels of fluorescence were detected in both the blank group and the Cy5.5-*p*-QM-1h-NLC treatment group. These results also indicated that CTX-*p*-QM-1h-NLC could increase the cellular uptake of *p*-QM-1h by targeting EGFR. However, the cellular uptake experiment involved direct interaction between nanomedicine and cells, lacking the in vivo “barrier and transport” process.

#### 2.6.2. In Vivo Targeting Study of CTX-p-QM-1h-NLC

In addition to the in vitro targeting study of CTX-*p*-QM-1h-NLC, we further conducted in vivo targeting studies of it. Quantitative analysis of CTX-*p*-QM-1h-NLC in tumors was performed using HPLC, as shown in Figure 7C. The content of *p*-QM-1h in tumors of mice treated with CTX-*p*-QM-1h-NLC reached its highest level at 1.5 h, which was about 2 times higher than that of mice treated with free *p*-QM-1h. Furthermore, qualitative analysis was conducted on mice treated with Cy5.5-CTX-*p*-QM-1h-NLC. As shown in Figure 7D, the tumor fluorescence images showed that the high fluorescence intensity was observed at around 0.75 and 1.5 h, indicating that *p*-QM-1h could effectively aggregate in the tumor.

The results of in vitro and in vivo experiments indicated that CTX-*p*-QM-1h-NLC could effectively target cancer cells and tumor tissues. In addition, the design of CTX-*p*-QM-1h-NLC could increase the solubility of free *p*-QM-1h, increase the aggregation of free *p*-QM-1h in tumors, and prolong drug release time, all of which could enhance drug efficacy, reduce drug dosage, decrease the passive targeting to normal tissues, and reduce systemic toxicity.

### 2.7. Safety Evaluation of CTX-p-QM-1h-NLC

In the development of anti-cancer drugs, the safety evaluation in mouse experiments is a key link connecting in vitro research and clinical trials. Based on the in vivo anti-tumor evaluation experiment (15 days), organ indices, serum indicators (aspartate transaminase (AST), alanine aminotransferase (ALT), blood urea nitrogen (BUN) and creatinine (CRE)) and histopathology (liver, spleen and kidney) were measured to preliminarily evaluate the short-term damage of drugs to normal organs.

In vivo anti-tumor activity in mice and blood biochemical indicators AST, ALT, BUN and CRE are the core components for evaluating drug safety and organ toxicity. AST and ALT can be likened to “sentinels” of liver toxicity; BUN and CRE can be likened to “sentinels” of nephrotoxicity.

**Figure 8:**
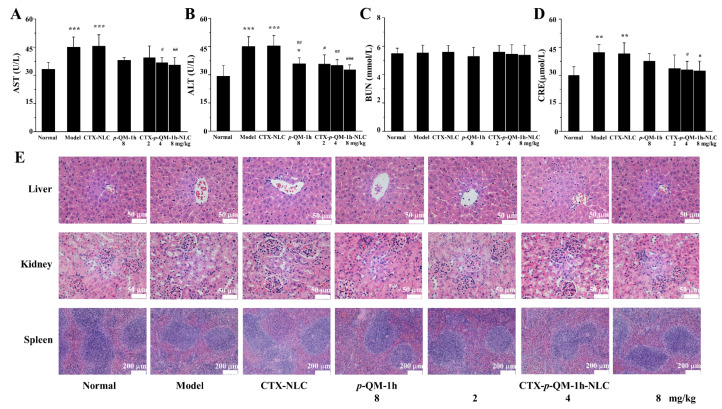
Effects of CTX-*p*-QM-1h-NLC on blood biochemical indicators and histopathological study related to tissue damage. (**A**) AST, (**B**) ALT, (**C**) BUN and (**D**) CRE. The experiment was performed at least in triplicate. Error bars represent mean ± SD. * *p* < 0.05, ** *p* < 0.01 and *** *p* < 0.001 compared with the normal group; ^#^ *p* < 0.05, ^##^ *p* < 0.01 and ^###^ *p* < 0.001 compared with the model group. (**E**) Representative histopathological images of liver, kidney and spleen (scale bars: liver 50 μm, kidney 50 μm, and spleen 200 μm).

As shown in Figure 8A,B, compared with normal mice, the AST and ALT levels in the model group or CTX-NLC group were significantly increased, indicating that the liver of the model group or CTX-NLC group was damaged. Compared with the AST level in the model group, the AST level in the 4 mg/kg or 8 mg/kg CTX-*p*-QM-1h-NLC treatment groups was significantly reduced, and there was no statistical difference in AST levels between the normal group and the CTX-*p*-QM-1h-NLC groups. Compared with the ALT level in the model group, the ALT levels in the *p*-QM-1h and CTX-*p*-QM-1h-NLC treatment groups were significantly reduced, and there was no statistical difference in ALT levels between the CTX-*p*-QM-1h-NLC treatment group and normal mice.

As shown in Figure 8C, there was no statistically significant difference in BUN levels among all groups of mice. However, as shown in Figure 8D, compared with the CRE level in the normal group, the CRE levels in the model and CTX-NLC groups increased; compared with the model group CRE, the CRE levels of the mice treated with 4 mg/kg and 8 mg/kg CTX-*p*-QM-1h-NLC were reduced.

These serum biochemical indicators (AST, ALT and CRE) indicated that CTX-*p*-QM-1h-NLC could effectively reduce liver and kidney damage caused by tumors during the experimental period. However, due to the short duration of the mouse experiment, no pathological changes were detected in the liver, spleen, and kidney of each experimental group (Figure 8E).

The organ index is a very important and fundamental toxicological and pharmacological evaluation index. As shown in Table 2, compared with normal mice, the organ indices of the liver, kidney, and spleen in the model group were significantly increased. For the liver organ index, CTX-*p*-QM-1h-NLC at 4 mg/kg or 8 mg/kg could significantly inhibit the increase in liver organ index caused by tumors; however, 8 mg/kg *p*-QM-1h did not significantly inhibit this index. For the kidney organ index, CTX-*p*-QM-1h-NLC at 4 mg/kg or 8 mg/kg could significantly inhibit the increase in the renal organ index caused by tumors. For the spleen organ index, tumors caused significant enlargement of the spleen in mice, with the organ index increasing from 0.0054 ± 0.00059 in normal mice to 0.022 ± 0.0036. CTX-*p*-QM-1h-NLC significantly reduced the spleen organ index, and intervention with 8 mg/kg *p*-QM-1h restored the spleen organ index to normal in mice. The above organ indices indicated that CTX-*p*-QM-1h-NLC could effectively inhibit organ damage to the liver, kidney, and spleen caused by tumors during the experimental period.

## 3. Material and Methods

### 3.1. Preparation of p-QM-1h-NLC

According to the literature method [21], a batch of *p*-QM-1h-NLC was prepared according to the quality of each lipid in Table S1. A 6 mg amount of *p*-QM-1h was dissolved in 12.73 mg of Miglyol 812N at 75 °C. The other lipids were dissolved in 5 mL methanol, and then mixed with Miglyol 812N solution containing *p*-QM-1h. The mixed solution was subjected to vacuum distillation to remove methanol at 80 rpm and 75 °C, under −0.01 MPa in a 100 mL round-bottom flask. The lipid film on the bottle wall was hydrated in 2 mL PBS at 80 rpm, 75 °C and −0.01 MPa for 20 min, and was shaken by ultrasound. Then, the 2 mL mixture was sonicated using an ultrasonic cell disruptor (Xiaomei Chaosheng, XM100DT, Kunshan, China) for 6 min at 25 kHz and 4 °C. After extruding through the 200 nm polycarbonate membrane of a liposome extruder, the resulting lipid solution was dialyzed in PBS (10 mM, pH 7.4) for 12 h (MWCO 1000 Da, Yuanye, Shanghai, China).

### 3.2. Preparation of CTX-p-QM-1h-NLC

The CTX antibody (1.0 molar equivalent) was mixed with Traut reagent (2-iminothiolane, 20 times molar equivalent) for sulfurization of free amino groups in PBS (10 mM, pH 8.0) at 4 °C. Sulfurized CTX antibodies were dialyzed to remove excess Traut reagent in PBS (10 mM, pH 7.4) at 4 °C overnight (MWCO 1000 Da). The SH (1.5 times molar equivalent) of sulfurized CTX antibody was reacted with maleimide (1.0 molar equivalent) on *p*-QM-1h-NLC at 4 °C for 24 h. The obtained CTX-*p*-QM-1h-NLC was dialyzed in PBS (10 mM, pH 7.4) for 12 h to remove excess sulfurized CTX antibodies (MWCO 3000 Da, Yuanye, Shanghai, China).

### 3.3. Measurement of Nano Features of p-QM-1h-NLC and CTX-p-QM-1h-NLC

The nanoparticle size, PDI and Zeta potential of *p*-QM-1h-NLC, CTX-*p*-QM-1h-NLC and CTX-NLC, and the stability of *p*-QM-1h-NLC were determined using Zetasizer Nano ZSE (Malvern, Worcestershire, UK). An integrated 4 mV He-Ne laser (λ = 633 nm) and scattering angle θ = 175° backscattering detection were set for DLS detection. Samples were dispersed in PBS (10 mM, pH 7.4) at 25 °C.

A 50 μL volume of *p*-QM-1h-NLC was mixed with 10 μL dimethyl sulfoxide and 20 μL Triton-X 100, and then vortexed for 30 s and allowed to stand for 30 min. Then, 320 μL acetonitrile was added to the above solution, and the mixture solution was vortexed and centrifuged at 12,000 *g* for 10 min. The supernatant was taken to determine the amount of *p*-QM-1h using HPLC for calculating EE and DLC.

EE% = (the amount of *p*-QM-1h encapsulated in nanoparticles/total *p*-QM-1h dosage) × 100%.

DLC% = (the amount of *p*-QM-1h encapsulated in nanoparticles/total mass of liposomes) × 100%.

### 3.4. FTIR Spectroscopy

CTX-p-QM-1h-NLC and CTX-NLC were freeze-dried using a freeze dryer (Scientz-10ND, Ningbo, China). Next, 150 mg KBr and 0.75 mg different sample (dry CTX-*p*-QM-1h-NLC, dry CTX-NLC, *p*-QM-1h, the physical mixture of *p*-QM-1h and liquid) were mixed, then ground and compressed. The sample was scanned using an FTIR spectrometer (Gangdong FTIR-850, Tianjin, China).

### 3.5. Measurement of Antibody Characteristics of CTX-p-QM-1h-NLC

The connection efficiency of CTX on the surface of CTX-*p*-QM-1h-NLC was determined by measuring the protein concentration of CTX using BCA assay kit (P0010, Beyotime, Shanghai, China). The connection efficiency was equal to the amount of CTX connected to the surface of CTX-*p*-QM-1h-NLC divided by the amount of CTX at the theoretical sites in CTX-*p*-QM-1h-NLC.

Protein integrity testing on CTX connected to the surface of CTX-*p*-QM-1h-NLC was performed. SDS-PAGE was used for primary structure analysis. After gel was stained with Coomassie brilliant blue, the bands of free CTX were compared with the bands of CTX connected on the surface of CTX-*p*-QM-1h-NLC.

The tertiary structure of CTX was analyzed using a Hitachi F-7000 fluorescence spectrophotometer (Hitachi, Japan), and the maximum emission wavelengths of free CTX, thiolated CTX, and CTX connected on surface of CTX-*p*-QM-1h-NLC were compared, as well as the effects of the denaturing agent Gdn·HCl on the maximum emission wavelength and fluorescence intensity.

### 3.6. In Vitro Drug Release

A dialysis bag (MWCO 10,000 Da, Yuanye, Shanghai, China) containing 0.5 mL of 2.48 mg/mL CTX-*p*-QM-1h-NLC solution was placed in 50 mL of PBS (10 mM, pH 7.4) solution containing 1% Tween 20 at 37 °C, and *p*-QM-1h was released for 48 h. During the experiment, 0.5 mL of release solution was taken out at different time points, and 0.5 mL of fresh release solution was added. The *p*-QM-1h concentration of the release solution was measured, and the cumulative release amount of *p*-QM-1h was calculated using high-performance liquid chromatography (HPLC, Agilent Technologies 1260 Infinity, CA, USA).

### 3.7. In Vitro Targeting Study in 4T1 Cells

#### 3.7.1. Cellular Uptake

Two milliliters of 1.5 × 10^5^ cells/mL 4T1 cells (TCM32, the cell bank of Chinese Academy of Sciences, China) was incubated in RPMI 1640 medium (Viva cell, Shanghai, China) containing 10% fetal bovine serum (Viva cell) and 1% antibiotic solution (C0222, Beyotime, Shanghai, China) in a 6-well plate for 24 h. Cells were incubated in fresh 1640 medium with 20 μM free *p*-QM-1h, *p*-QM-1h-NLC or CTX-*p*-QM-1h-NLC (pretreated with/without CTX) for different durations (0, 0.5, 1, 2, 3,6 h). After cells were washed with PBS twice, 0.8 mL MeOH was added to extract *p*-QM-1h at 4 °C for 10 h. Then, the suspension was centrifuged at 10,000 *g* and 4 °C for 5 min. The absorbance of 200 μL supernatant was recorded at 348 nm using a microplate reader (Tecan, Freedom EVO-100, Grödig, Austria).

#### 3.7.2. Flow Cytometry

Cy5.5-CTX-*p*-QM-1h-NLC or Cy5.5-*p*-QM-1h-NLC was prepared according to the preparation method of CTX-*p*-QM-1h-NLC mentioned above, except that 1 mg DSPE-PEG2000 in Table S1 was replaced with 1 mg Cy5.5-DSPE-PEG2000.

Two milliliters of 2.0 × 10^5^ cells/mL 4T1 cells was incubated in a 6-well plate for 24 h. After incubating with fresh 1640 medium containing 200 μL Cy5.5-CTX-*p*-QM-1h-NLC or Cy5.5-*p*-QM-1h-NLC for 3 h, cells were washed with PBS twice, resuspended in PBS, and detected using flow cytometry (Cytek^®^ Aurora/Northern Lights^TM^, Fremont, CA, USA).

### 3.8. In Vitro Anti-Cancer Evaluation

#### 3.8.1. Anti-Proliferative Activity

One hundred microliters of 1.0 × 10^4^ cells/mL 4T1 cells was incubated in a 96-well plate for 24 h. After cells were incubated with fresh 1640 medium containing different concentrations of CTX-*p*-QM-1h-NLC, CTX-NLC, free *p*-QM-1h or CTX for 48 h, 10 μL MTT solution (5 mg/mL, C0009, Beyotime, Shanghai, China) was added and then incubated in the dark for 4 h. The absorbance was recorded at 570 nm using a microplate reader (Tecan, Freedom EVO-100, Grödig, Austria).

#### 3.8.2. Determination of ROS

Two milliliters of 2.0 × 10^5^ cells/mL 4T1 cells was incubated in a 6-well plate for 24 h. After incubation with fresh 1640 medium containing 200 μL CTX-*p*-QM-1h-NLC or *p*-QM-1h for 6 h, cells were washed with PBS twice and stained with DCFH-DA solution (3 μM, S1105, Beyotime, Shanghai, China) in the dark for 30 min. Then, cells were washed twice with PBS, resuspended in PBS, and detected using flow cytometry (Cytek^®^ Aurora/Northern Lights^TM^, Fremont, CA, USA).

#### 3.8.3. Determination of MMP

Two milliliters of 2.0 × 10^5^ cells/mL 4T1 cells was incubated in a 6-well plate for 24 h. After incubation with fresh 1640 medium containing 200 μL CTX-*p*-QM-1h-NLC or *p*-QM-1h for 15 h, cells were washed with PBS twice and stained with Rhodamine 123 solution (5 μM, C2007, Beyotime, Shanghai, China) in the dark for 30 min. Then, cells were washed twice with PBS, resuspended in PBS, and detected using flow cytometry (Cytek^®^ Aurora/Northern Lights^TM^, Fremont, CA, USA).

#### 3.8.4. Cell Apoptosis Assay

Two milliliters of 2.0 × 10^5^ cells/mL 4T1 cells was incubated in a 6-well plate for 24 h. After incubation with fresh 1640 medium containing 200 μL CTX-*p*-QM-1h-NLC or *p*-QM-1h for 24 h, cells were washed with PBS twice. Then, cells were stained and determined using the commercial reagent kit (Annexin V-FITC apoptosis detection kit, C1062, Beyotime, Shanghai, China) according to the product instructions.

### 3.9. In Vivo Anti-Tumor Evaluation

The Balb/c mice (8 weeks old, female, 18–20 g, *n* = 35) were purchased from Pengyue Experimental Animal Breeding Co., Ltd (Jinan, China) and kept in the SPF animal room. All animal studies were conducted under the National Institute Guide for the Care and Use of Laboratory Animals, and were approved by the Ethics Committee of Liaocheng University (2022111013).

A 0.15 mL volume of 4T1 cells at a density of 1 × 10^7^ cells/mL was seeded subcutaneously under the left axilla of Balb/c mice. On the third day, obvious tumor mass could be touched. Tumor-bearing mice were randomly divided into 6 groups, with 5 mice in each group, and drugs were injected through the tail vein daily. In addition, there was a normal mouse group without tumors (5 mice). The groups comprised the (1) Normal group; (2) Model group; (3) CTX-NLC group; (4) 8 mg/kg *p*-QM-1h group (dissolved in 5% anhydrous ethanol and 5% polyoxyethylene (35) castor oil of saline solution); (5) 2 mg/kg CTX-*p*-QM-1h-NLC group; (6) 4 mg/kg CTX-*p*-QM-1h-NLC group; (7) 8 mg/kg CTX-*p*-QM-1h-NLC group. The injection volume (80 μL) of the CTX-NLC group was equivalent to that of the 8 mg/kg CTX-*p*-QM-1h-NLC group (80 μL), and the CTX-NLC group was compared with the 8 mg/kg CTX-*p*-QM-1h-NLC group. All mice were sacrificed on the 15th day, blood was collected from the eyeballs, and various organs were frozen in liquid nitrogen or soaked in 4% paraformaldehyde fix solution (P0099, Beyotime, Shanghai, China).

### 3.10. Serum Index Determination

The serum biochemical indicators related to liver and kidney injury were measured using commercial reagent kits according to the product instructions. The commercial reagent kits included the aspartate transaminase (AST)/glutamic oxaloacetic transaminase (GOT) activity detection kit (E2023, Applygen, Beijing, China), the alanine aminotransferase (ALT)/glutamate pyruvate transaminase (GPT) activity detection kit (E2021, Applygen, Beijing, China), the urea (BUN) assay kit (C013-1-1, Nanjing Jiancheng, Nanjing, China), and the creatinine (CRE) assay kit (C011-2-1, Nanjing Jiancheng, Nanjing, China).

### 3.11. Hematoxylin Eosin Staining

The tumor, liver, spleen or kidney was fixed with 4% paraformaldehyde fix solution (P0099, Beyotime, Shanghai, China), embedded in paraffin, sliced, and dewaxed. After staining with hematoxylin eosin (C0105, Beyotime, Shanghai, China), the sample was dehydrated, transparent, and sealed, and images were taken using an Olympus microscope (BX53 + DP80, Oberkochen, Germany).

### 3.12. In Vivo Targeting Study in Mice

On the 15th day of subcutaneous injection of 4T1 cells into mice, 80 μL of Cy5.5-CTX-*p*-QM-1h-NLC was injected into the tail vein of tumor-bearing mice. At 0, 0.75, 1.5, 3, 6, 12, and 24 h of tail vein injection, mice were euthanized and tumors were collected for bioluminescence imaging using IVIS Lumina LT Series III (PerkinElmer, Ruifudi, Springfield, IL, USA).

On the 15th day of subcutaneous injection of 4T1 cells into mice, 80 μL of 2.49 mg/mL CTX-*p*-QM-1h-NLC (8 mg/kg/day) or *p*-QM-1h (8 mg/kg/day) was injected into the tail vein of tumor-bearing mice. After 0, 0.375, 0.75, 1.5, 3, 6, 12 and 24 h of tail vein injection, the mice were euthanized, and various organs were collected. *p*-QM-1h was extracted three times from the tissue homogenate solution with ethyl acetate. The organic solutions were combined, and were concentrated under reduced pressure. The concentrate was dissolved in methanol and filtered. The concentration of *p*-QM-1h was analyzed using HPLC (Agilent Technologies 1260 Infinity, CA, USA). (Eluent, methanol: H_2_O = 90:10; flow rate, 1 mL/min; λ, 348 nm; retention time, 14.903 min; injection volume, 10 μL; linear relationship: y = 13952x + 10.367, x mg/mL, y peak area)

### 3.13. Western Blot

Following the general procedure of Western blot, the expressions of TrxR, Bax and caspase-3 in mouse tumor tissues were determined. The primary antibodies used in the experiments were as follows: TrxR (bs-8299R, Bioss, Beijing, China), Bax (bsm-52316R, Bioss, Beijing, China), Caspase-3 (bsm-61071R, Bioss, Beijing, China) and β-actin (GB15003, Servicebio, Wuhan, China). The secondary antibodies were as follows: HRP-labeled goat anti-rabbit IgG (H + L) (A0208, Beyotime, Shanghai, China) and HRP-labeled goat anti-mouse IgG (H + L) (A0216, Beyotime, Shanghai, China).

### 3.14. Statistical Analysis

All experiments were conducted in at least three independent replicates. The number of mice in the experiment was 5. All data were presented as mean ± standard deviation. Statistical significance analysis of the data was performed using a one-way analysis of variance test and Dunnett test in GraphPad Prism software (Prism 5 for Windows).

## 4. Conclusions

*p*-QM-1h has excellent anticancer activity, but as an organic small molecule, it has low solubility in physiological environments and requires a high dosage. In this study, we loaded *p*-QM-1h into NLC and modified with CTX targeting EGFR on the surface of *p*-QM-1h-NLC to construct CTX-*p*-QM-1h-NLC, which was uniform, well dispersed, and had moderate stability. We chose Miglyol 812N as the liquid lipid, which effectively increased the solubility of p-QM-1h (from 14.54 μg/mL to 2.49 mg/mL) and had a high EE. Immune characteristics and cellular uptake showed that CTX on the surface of CTX-*p*-QM-1h-NLC was structurally intact and functionally preserved. The design of CTX-*p*-QM-1h-NLC did not affect the anti-proliferative activity of *p*-QM-1h in 4T1 cells, and could increase the ROS level in 4T1 cells, leading to MMP breakdown and inducing cell apoptosis. Moreover, anti-tumor experiments in mice have shown that CTX-*p*-QM-1h-NLC could effectively inhibit tumor growth in mice in a dose-dependent manner. Additionally, the inhibitory effect of 2 mg/kg CTX-*p*-QM-1h-NLC on tumors was similar to that of the 8 mg/kg free *p*-QM-1h, effectively improving the efficacy of *p*-QM-1h and reducing the dosage of *p*-QM-1h used. The intervention with CTX-*p*-QM-1h-NLC in tumor-bearing mice could effectively inhibit the expression of TrxR and increase the expressions of Bax and Caspase-3, which were consistent with the increase in ROS levels and the initiation of cell apoptosis in 4T1 cells. Further, in vitro and in vivo targeting experiments and safety evaluations have shown that CTX-*p*-QM-1h-NLC has a certain targeting ability and in vivo safety in the short term.

In conclusion, the construction of CTX-*p*-QM-1h-NLC could effectively increase the solubility of *p*-QM-1h, increase the enrichment of *p*-QM-1h in lesion tumor tissue, enhance the efficacy of *p*-QM-1h, and reduce the dosage of *p*-QM-1h, thus improving the safety of the drug, which means that this was a valuable study and deserves further investigation. In further study, we will conduct more detailed studies on the optimization of NLC construction, the improvement of nanomedicine targeting, pharmacokinetics of drugs, and systematic toxicology evaluation (acute and chronic) and so on.

## Figures and Tables

**Table 1:** Characterization of *p*-QM-1h-NLC, CTX-*p*-QM-1h-NLC and CTX-NLC.

NPs	Size (d.nm)	PDI	Zeta (mV)	EE (%)	DLC (%)	CE (%)
*p*-QM-1h-NLC	136.77 ± 0.52	0.125 ± 0.001	−20.07 ± 0.67	82.80 ± 1.48	7.37 ± 0.13	/
CTX-*p*-QM-1h-NLC	150.53 ± 0.68	0.123 ± 0.012	−19.07 ± 0.25	/	/	87.98 ± 3.68
CTX-NLC	132.3 ± 0.85	0.128 ± 0.016	−17.37 ± 0.29	/	/	/

**Table 2:** Organ index of the liver, kidney and spleen.

Groups	Liver	Kidney	Spleen
Normal	0.044 ± 0.0046	0.012 ± 0.0010	0.0054 ± 0.00059
Model	0.062 ± 0.0041 ***	0.015 ± 0.0013 ***	0.022 ± 0.0036 ***
CTX-NLC	0.053 ± 0.0033 *	0.014 ± 0.0003	0.020 ± 0.0015 ***
*p*-QM-1h 8 mg/kg	0.054 ± 0.0057 **	0.013 ± 0.0011 ^##^	0.018 ± 0.0051 ***
CTX-*p*-QM-1h-NLC 2 mg/kg	0.054 ± 0.0028 *	0.013 ± 0.0003 ^#^	0.014 ± 0.0018 ***, ^###^
CTX- *p*-QM-1h-NLC 4 mg/kg	0.050 ± 0.0049 ^##^	0.013 ± 0.0008 ^###^	0.014 ± 0.0017 ***, ^###^
CTX- *p*-QM-1h-NLC 8 mg/kg	0.050 ± 0.0089 ^##^	0.012 ± 0.0007 ^###^	0.0065 ± 0.0029 ^###^

* *p* < 0.05, ** *p* < 0.01 and *** *p* < 0.001, compared with the normal group; ^#^ *p* < 0.05, ^##^ *p* < 0.01 and ^###^ *p* < 0.001, compared with the model group.

## Data Availability

Data will be provided by the corresponding author as required.

## Supplementary Materials

The following supporting information can be downloaded at: https://www.mdpi.com/article/10.3390/ijms27083674/s1.
