# Supplementary material for: Enhanced Anti-Tumor Activity of Cetuximab-Modified Nanostructured Lipid Carriers Loaded with Para-Quinone Methide Derivative p-QM-1h
Source: Int J Mol Sci. 2026 Apr 20;27(8):3674. doi: 10.3390/ijms27083674 (PMC13115955; doi:10.3390/ijms27083674)
Supplement: Supplementary file 1 [file ijms-27-03674-s001.zip › ijms-4185995-supplementary.pdf]

Enhanced Anti-Tumor Activity of Cetuximab-Modified Nanostructured Lipid Carriers Loaded with *Para*-Quinone Methides Derivatives *p*-QM-**1h**

Xuanze Lyu, Meijia Liu, Hanqing Li, Junyi Cui, Jie Yang*, Guoyun Liu*

State Key Laboratory of Macromolecular Drugs and Large-scale Preparation, Shandong Key Laboratory of Applied Technology for Protein and Peptide Drugs, School of Pharmaceutical Sciences and Food Engineering, Liaocheng University, 1 Hunan Street, Liaocheng, 252059, Shandong, China

Supporting Information

*Corresponding author

E-mail: yangjie3@lcu.edu.cn; liuguoyun@lcu.edu.cn.

Table S1. Lipid components of *p*-QM-**1h** loaded nanostructured lipid carriers (NLCs).

	Miglyol 812N	DSPC	Cholesterol	DSPE-PEG2000	DSPE-PEG2000-MAL
**MW**/g/mol	505	790	386.65	2805.50	2893.00
**n**/mmol	0.0252	0.0378	0.0378	0.0052	0.0001
**mole ratio**	23.75	35.63	35.63	4.90	0.09
**m**/mg	12.73	29.87	14.62	14.59	0.31

Various lipids for prepare of *p*-QM-**1h**, as well as the amount, molar ratio and mass of each lipid.

Table S2. The solubility of *p*-QM-**1h** in PBS or NLC containing different liquid lipids.

	Free *p*-QM-**1h**	Oleic acid	MCT	Castor oil	Miglyol 812N
Solubility	14.54±0.60 μg/mL	0.34 mg/mL	2.63 mg/mL	2.42 mg/mL	2.49 mg/mL

Compare the solubility of *p*-QM-**1h** in PBS or NLC containing different liquid lipids.

Table S3. Comparison of size, PDI, Zeta and EE% of *p*-QM-**1h** NLC prepared using different liquid lipids.

	Oleic acid[a]	MCT	Castor oil[a]	Miglyol 812N
**Size (d. nm)**	139.2	131.0	127.2	136.77
**PDI**	0.190	0.138	0.159	0.125
**Zeta (mV)**	-13.6	-10.3	-7.17	-20.07
**EE%**	11.33	87.67	80.67	82.80
**Cost(¥/mL)**	19	1	0.08	0.654

a There is precipitation in the NLC solution prepared using oleic acid or castor oil as liquid lipids.

The NLC solution prepared using oleic acid as liquid lipid precipitates after dialysis. The NLC solution prepared using Castor oil as liquid lipid precipitates after 24 hours of storage at 4 °C.

Table S4. The maximum emission wave length of free CTX, sulfurized CTX, CTX-*p*-QM-**1h**-NLC in the presence or absence of guanidine hydrochloride (Gdn.HCl).

Num	The maximum emission wave length (nm)
CTX	346.67±0.12
CTX + Gdn.HCl	355.67±0.31
sulfurized CTX	346.27±0.23
sulfurized CTX + Gdn.HCl	356.2±0.60
CTX-*p*-QM-**1h**-NLC	346.0±0.20
CTX-*p*-QM-**1h**-NLC + Gdn.HCl	356.2±0.42

The dosage of CTX injected into the tail vein of mice:

(1)

(0.00031 g / 2893 g/mol) × 2756 g/mol × 88% x (80 μL/2 mL) = 0.0104 mg

(2)

$A = K \times W^{2/3} = 9.1 \times W^{2/3} /10000 = 9.1 \times 20^{2/3} = 0.0067\ m^2$ (A m^2, W g)

(3)

$0.0104\ mg / 0.0067\ m^2 = 1.55\ mg/m^2$

Table S5. Effects on cell viability in the 4T1 cells.

Num	CTX-*p*-QM-**1h**-NLC	*p*-QM-**1h**	CTX-NLC	CTX
IC_{50} (μM)	3.96±0.02	4.05±0.03	>100	>100

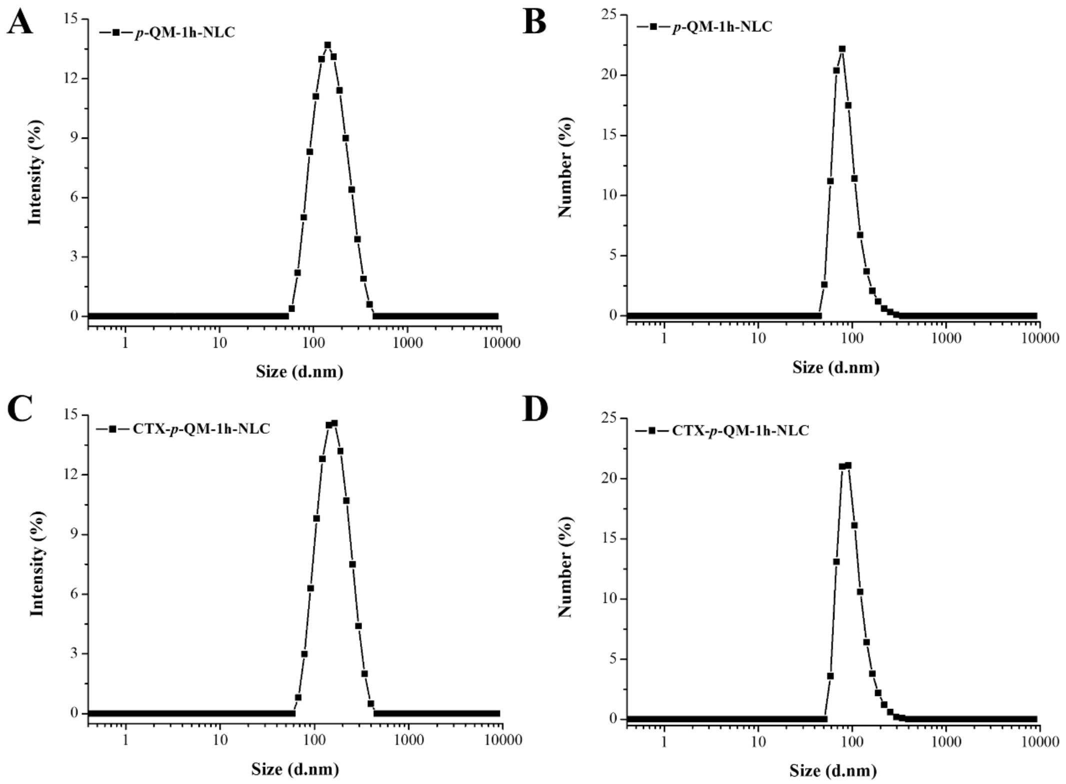

Figure S1. The particle size distribution (intensity and number intensity) of *p*-QM-**1h**-NLC and CTX- *p*-QM-**1h**-NLC.

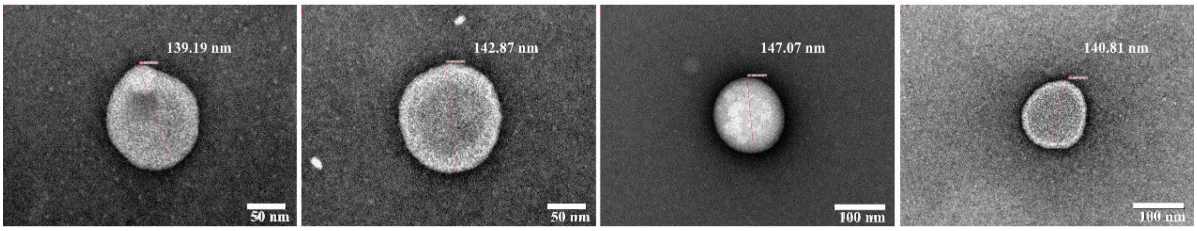

Figure S2. Representative TEM images of CTX-*p*-QM-**1h**-NLC.

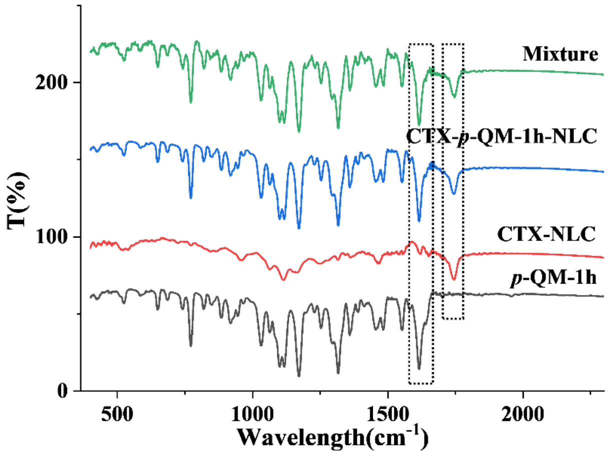

Figure S3. FTIR spectra of *p*-QM-**1h**, CTX-NLC, CTX-*p*-QM-**1h**-NLC and the physical mixture of *p*-QM-**1h**, CTX and liquids which were mixed in the same ratio as the preparation of nanomedicine.
